# A systematic review of the impact of brain tumours on risk of motor vehicle crashes

**DOI:** 10.1007/s11060-024-04586-6

**Published:** 2024-02-06

**Authors:** Sophie Tran, Adam Lapidus, Andrew Neal, Katherine B. Peters, Lucy Gately, Malaka Ameratunga

**Affiliations:** 1 Department of Medical Oncology, Alfred Health, Melbourne, VIC Australia; 2 Department of Neuroscience, Monash University, Melbourne, VIC Australia; 3 Central Clinical School, Monash University, Melbourne, VIC Australia; 4 Walter and Eliza Hall Institute for Medical Research, The University of Melbourne, Melbourne, VIC Australia; 5 Department of Neurosurgery, Duke University, Durham, NC USA

**Keywords:** Driving, Brain tumour, Motor vehicle accidents, Motor vehicle crashes

## Abstract

**Purpose:**

Brain tumours are associated with neurocognitive impairments that are important for safe driving. Driving is vital to maintaining patient autonomy, despite this there is limited research on driving capacity amongst patients with brain tumours. The purpose of this review is to examine MVC risk in patients with brain tumours to inform development of clearer driving guidelines.

**Methods:**

A systematic review was performed using Medline and EMBASE. Observational studies were included. The outcome of interest was MVC or measured risk of MVC in patients with benign or malignant brain tumours. Descriptive analysis and synthesis without meta-analysis were used to summarise findings. A narrative review of driving guidelines from Australia, United Kingdom and Canada was completed.

**Results:**

Three studies were included in this review. One cohort study, one cross-sectional study and one case–control study were included (19,135 participants) across United States and Finland. One study evaluated the incidence of MVC in brain tumour patients, revealing no difference in MVC rates. Two studies measured MVC risk using driving simulation and cognitive testing. Patients found at higher risk of MVC had greater degrees of memory and visual attention impairments. However, predictive patient and tumour characteristics of MVC risk were heterogeneous across studies. Overall, driving guidelines had clear recommendations on selected conditions like seizures but were vague surrounding neurocognitive deficits.

**Conclusion:**

Limited data exists regarding driving behaviour and MVC incidence in brain tumour patients. Existing guidelines inadequately address neurocognitive complexities in this group. Future studies evaluating real-world data is required to inform development of more applicable driving guidelines.

**Systematic review registration number:**

PROSPERO 2023 CRD42023434608.

**Supplementary Information:**

The online version contains supplementary material available at 10.1007/s11060-024-04586-6.

## Background

Driving is a complex task that requires intact neurocognitive skills including higher executive function, complex attention, visuospatial and motor function. Primary and metastatic brain tumours are associated with high morbidity and mortality, often requiring multimodality treatments including surgical resection, systemic and radiation therapy. As such, patients may be symptomatic from both disease and treatment. Seizures are a common complication of both primary and metastatic brain tumours and a major determinant of driving restrictions [1]. Approximately 80–90% of patients with diffuse gliomas will experience at least one seizure during their disease course [2, 3].

Motor vehicle crashes are a global public health issue. In Australia, there were 61,483 transport injuries (including car-occupied, motorcyclists, pedal cyclists and pedestrians) with 1,394 of these resulting in death between 2021 to 2022 [4]. In the US, there were roughly 36,355 deaths (1.21 death per registered motor vehicle related to motor vehicle crashes in 2019) [5]. Treating providers of patients with brain tumours have both a legal and ethical obligation in assessing patients’ fitness to drive. Despite this, they often have no formal training and lack resources and tools beyond guidelines that are formed on limited evidence base with utility limited to specific situational scenarios, such as seizures [6–8].

Driving is a crucial component of maintaining independence and quality of life. A longitudinal study revealed that patients with primary brain tumours attributed their loss of personal autonomy and day-to-day life problems to their inability to drive [9]. However, there remains little research exploring the driving capacity and implications of patients with brain tumours.

To our knowledge, there has not been a systematic review that has evaluated the relationship of driving with brain tumours and incidence of motor vehicle crashes (MVC). The aim of this study is to examine whether brain tumour patients are at higher risk of MVC. Addressing this question will inform the development of more comprehensive and clinically applicable driving guidelines for clinicians and patients.

## Method

**Fig. 1:**
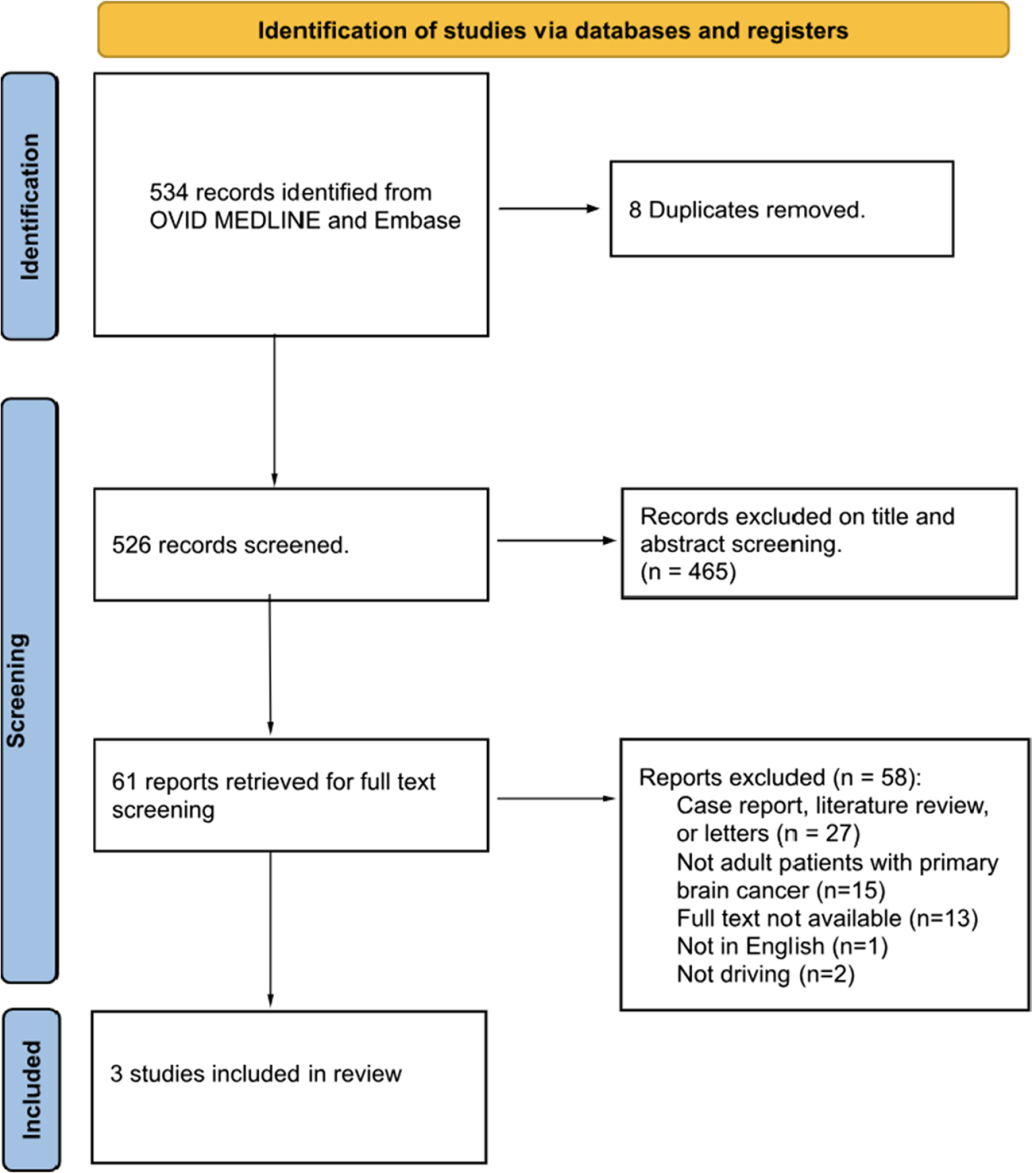
PRISMA flow diagram

This study meets the requirements of the Preferred Reporting Items for Systematic Reviews and Meta-analyses (PRISMA) statement [10] (Systematic review registration number: PROSPERO 2023 CRD42023434608) (Fig. 1).

### Eligibility criteria

Observational studies that were eligible for this systematic review included cohort studies, case-control studies and retrospective studies. The inclusion criteria were observational studies that were: reported in a peer-reviewed journal; the outcome of interest were MVC or measured risk of MVC by assessment (such as driving simulation, cognitive battery); and the exposure was driving with brain tumours including benign and malignant (primary and metastatic). The exclusion criteria were: case reports and case series; non-English studies; and studies of adults with history of paediatric intracranial tumours.

### Search Strategy

Medline and Embase databases were searched using keywords related to brain tumour and MVC. Two reviewers (ST and AL) developed the search strategy. The search was developed and conducted in March 2023. The full search strategy and details of keywords used in the Medline database are reported in the Supplementary material. Time restriction was applied from 1974 to 7 March 2023**.**

### Study selection

All eligible studies were compiled following the initial screening and duplicates removed. Two independent reviewers (ST and AL) reviewed all potential studies in three stages. Title and abstract screening of all records was performed initially. During this stage, if the eligibility of the study could not be determined, the article was retrieved for a full text review. Following title and abstract screening, full text articles of remaining records were screened. Additionally, the reference lists of included studies were also screened for further relevant articles that may have been missed on initial screening. Disagreements were resolved through discussions between reviewers.

Only national driving guidelines from English-speaking countries were included in this review. Guidelines from Australia, United Kingdom and Canada were accessed from each region’s driving board – Austroads and the National Transport Commission, Driver and Vehicle Licensing Agency and Canadian Medical Association respectively.

### Data extraction

Data extracted from studies included: Study details (journal, publication year, study design); Patient details (age, sex, demographics, tumour location, tumour histopathology, symptoms of brain tumour, treatment type if applicable); Assessments of risk of MVC (type of cognitive assessments, driving simulation scenarios); and results (incidence of MVC, measured risk of MVC). Extracted data was corroborated by two reviewers (ST and AL). Study characteristics and interventions were tabulated for synthesis and comparison.

### Evaluation of quality of studies

Quality of studies was independently assessed by two reviewers (ST and AL) using the Newcastle-Ottawa Scale (NOS) [11]. NOS is based on a star-scoring system that assesses studies on three domains – selection, comparability and outcome. Each study was scored from zero to nine stars. Studies rating 0 to 2 stars were considered to be poor, 3 to 5 fair and 6 to 9 high.

### Data analysis

Due to the heterogeneity of study designs and outcome measures; a meta-analysis was not performed. Descriptive analysis and synthesis without meta-analysis were used to summarise findings. A narrative review of international driving guidelines was made.

## Results

### Study selection

The literature search identified 534 articles, of which 8 were duplicates and removed. The remaining 526 record abstracts and titles were screened with 465 records found to be irrelevant to the study question and thus excluded. Full-text reviews were undertaken of the remaining 61 records with 3 studies meeting all inclusion criteria and were included in the analysis. Among the 58 records excluded, 15 did not include adult patients with brain tumours, 13 did not have full texts available, two were not related to driving, one was not in English and 27 were case reports, literature reviews or letters to the editor.

### Characteristics of studies included

The three eligible studies were published between 2018 and 2022 and included 19,135 participants from two countries, Finland and United States. The median age of participants was 54.3 years. All studies were conducted on both sexes.

The three studies included by Huuskonen et al., Mansur et al., and Estevis et al., all had different study designs; involving a cohort study, case-control study and cross-sectional study respectively (Table 1). Huuskonen et al., measured the incidence of MVC using a national road traffic safety database. Mansur et al., used a driving simulation test as a measure of MVC risk. Estevis et al., used the Cognitive Behavioural Driver’s Inventory (CBDI) as a surrogate marker of driving ability.

**Table 1:** Summary of included studies

Author (year)	Study design and aim	Study period	Study Population	Method	Findings	Limitations
Huuskonen 2022	Cohort study Assessed road traffic accident risk in cancer cohort compared to non-cancer cohort	2013–2017 Median follow up time: 34 months	**Cancer cohort:** Cancer patients treated at Turku University Hospital **Primary brain tumours *n* = 154** **Control cohort (*n* = 6334):** Patients with acute appendicitis, cholecystitis or actinic keratosis treated at same hospital	RTAs were identified from insurance claim database of the Finnish Motor Insurers’ Centre. Primary endpoint: first RTA leading to injury (mild to fatal) Secondary endpoint: RTA leading to vehicle damage only, without injuries	**Primary brain tumour population** 1’ – no events in this group 2’ – no events in this group RTA risk overall not increased in any of the nine cancers of interest	Observational study Sample size of primary brain tumour is small. 66% of patients with primary brain tumours were excluded from study for not having a driving license
Mansur 2018	Case–control study Aim: Identify self-reported driving habits and behaviours. To characterise driving performance of patients with brain tumours. Identify cognitive deficits associated with poorer driving performance	Not reported	**Brain tumour cohort:** Adults patients with diagnosis of a brain tumour treated at Canadian hospital **Control cohort:** Healthy patients recruited from the community with no prior neurological or psychiatric illness Part 1: 84 participants Part 2: Cancer cohort *n* = 13 Control cohort *n* = 13	**Part 1:** Self-report surveys a) Driving Habits Questionnaire b) American Academy of Neurology Patient Questionnaire	**Self-report surveys** - More than 50% self-rated driving quality as ‘’good’ and more than 33% self-rated ‘excellent’ - 40% of patients had at least one collision since diagnosis	Small sample size with heterogeneous population. Majority of brain tumour cohort had benign tumours. Driving simulation task cannot model real-world driving. No single cognitive testing can predict driving performance with adequate sensitivity and specificity.
				**Part 2:** 2a) Driving simulator with two scenarios i) City Driving – various conditions including straight driving and turns with oncoming traffic ii) Bus following task – following a lead vehicle with various speed. 2b) Cognitive testing post driving simulation – Cognitive battery Control cohort: MoCA testing added to cognitive testing.	**Driving Simulator** i) City Driving Scenario Brain tumour cohort had significantly greater variability in speed than control group (U = 57, *p* < 0.05 ii) Bus Following task Brain tumour patients committed twice number of speed exceedances as control group (U = 41, *p* < 0.05) No significant difference on number of collisions in both scenarios **Cognitive testing** - Comparable frontal lobe function - Higher HADS anxiety score amongst brain tumour patients, (U = 30, *p* < 0.05)	
					**Correlation data** - Greater time since diagnosis positively correlated with increased number of total errors in City Driving (r_x_ = -0.798, *p* < 0.05) - Tumour size, tumour location, cognitive tests did not correlate with driving performance	
Estevis 2019	Retrospective cross-sectional study Identify cognitive, demographic and clinical characteristics of patients with brain tumours that are associated with driving safety	2011–2016	*N* = 64 Patients with primary brain tumours referred for clinical driving evaluation between at the Neuropsychology Service at the University of Texas MD Anderson Cancer Centre were identified	**Cognitive Behavioural Driver’s Inventory (CBDI)** - 4 computerised and 3 paper-and-pencil tasks yielding 28 performance measures to form General Driver’s Index (GDI28) - Patients were classified as Pass, Borderline or Fail	- 69% passed CBDI, 31% did not pass **Characteristics associated with Non-Passing group (Borderline or Fail)** - Older age – 62.6 years vs 49 years, t[62] = 3.39, *P* = 0.001 - Temporal lobe tumours (60% vs 25%) - Higher tumour grade – WHO Grade IV (95% vs 63%, *P* = 0.032) **Regression analysis** - Age is best predictor of CBDI accounting for 17% of variance	Small sample size CBDI is a proxy assessment and does not equate to on-the-road ability Referral bias, patients with relatively preserved functional status with relatively longer survival time since diagnosis particularly as majority had high grade malignant gliomas.

All studies were rated between five to six stars on the NOS and considered to be fair quality (Table 2). Estevis’ study performed the poorest in selection bias assessment and comparability. Regarding Huuskonen and Mansur’s studies, weaknesses concerned the selection of participants (outcome of interest not present at start of study, selection of controls) and outcome (follow-up not long enough for outcomes to occur and non-response rate).

**Table 2:** Quality assessment of studies using a modified Newcastle-Ottawa scale for assessing studies in the systematic review of road traffic accidents in patients with brain tumours

Study ID	Selection (4)				Comparability (2)	Outcome (3)			Total score (9⋆)	Quality of Assessment
	Representativeness of exposed cohort (⋆)	Selection of non-exposed cohort (⋆)	Ascertainment of exposure (⋆)	Demonstration outcome of interest not present at start of study	Comparability of cohorts on the basis of design or analysis (⋆⋆)	Assessment of outcome (⋆)	Was follow up long enough for outcomes to occur	Adequacy of follow up of cohorts		
Huuskonen 2022 (cohort)	⋆	⋆	⋆	0	⋆ Study adjusted for tumour type, sex	⋆	0	⋆	6⋆	Fair
Study ID	Selection Bias Assessment (4)				Comparability (2)	Outcome (3)			Total score (9⋆)	Quality of assessment
	Is the case definition adequate?	Representativeness of the cases	Selection of controls	Definition of controls	Comparability of cases and controls on the basis of design or analysis	Assessment of exposure	Same method of ascertainment for cases and controls	Non-response rate		
Mansur 2018 (case–control)	⋆	⋆	0	⋆	⋆ Age & sex matched between control and brain tumours	⋆	⋆	0	6⋆	Fair
Study ID	Selection Bias Assessment (5)				Comparability (1)	Outcome (3)			Total score (9⋆)	Quality of assessment
	Representativeness of sample (2)	Sample size	Non-respondents	Ascertainment of exposure	Confounding factors are controlled	Assessment of the outcome (2)	Statistical Test			
Estevis 2019 (cross-sectional)	⋆	0	⋆	0	0	⋆⋆ (record linkage)	⋆		5⋆	Fair

Studies rating 0–2 (poor quality), 3–5 (fair quality), 6–9 (good/high quality)

### Narrative synthesis

#### Huuskonen et al. (2022) [12]

The Finnish cohort study reviewed the incidence of MVC in a cancer cohort compared to a non-cancer cohort living in Southwest Finland from a nationwide motor registry. Among the 12,651 cancer and 6334 control patients included in the study, patients were divided into nine tumour streams. There were 452 patients with primary brain tumours identified, however only 154 patients were included as 66% were excluded due to license suspension. Though, the study has not specified the reason for license suspension, 40% of this group had experienced seizures. The primary endpoint was the first MVC leading to any injury and the secondary endpoint was MVC without injuries. Only MVCs of which patients were at-fault were included in the study. At a median follow-up of 53 months for control and 34 months for the cancer cohort, there were no events in the primary brain tumour cohort.. Overall, no significant difference in MVC risk was observed in the whole cancer group compared to controls (HR 0.89, [0.65–1.21]).

#### Mansur et al. (2018) [13]

This case-control study assessed MVC risk using a driving simulation and cognitive assessment. A self-reporting questionnaire on driving behaviours was also administered to 86 patients with brain tumours. The most common brain tumour types were pituitary adenomas (31%) and meningiomas (31%). Only seven patients had high grade malignant brain tumours. Most participants (70.9%) had surgical intervention, with the median time from surgery to survey completion being 36.3 months. Although approximately 40% of participants self-reported at least one collision since diagnosis, only one patient reported concerns with their driving behaviours, and 20% of participants limited their amount of driving from brain tumour diagnosis.

In the second part of the study, 26 participants (*n* = 13 brain tumour, *n* = 13 health controls) undertook the driving simulation and cognitive testing. The driving simulation included two experimental scenarios. The City Driving scenario involved driving in varying traffic conditions to assess ability to process and cope under higher cognitive demand. The ‘Bus following task’ required participants to follow a lead vehicle with changing speeds to assess attention and on-road adaptive ability. Brain tumour participants had significantly greater variability in speed than the control group (U = 57, *p* < 0.05) and twice the number of speed exceedances (U = 41, *p* < 0.95). There were no significant differences in the number of collisions in both scenarios. Tumour size and location did not correlate with driving performance. There was comparable executive function and visual attention between both groups, noting five patients (38%) had frontal lobe gliomas in the brain tumour group.

#### Estevis et al. (2019) [14]

This cross-sectional study retrospectively reviewed 64 patients with primary brain tumours who were referred to the Neuropsychology Service at the University of Texas for a clinical driving assessment between 2011 and 2016. The Cognitive Behavioural Driver’s Inventory (CBDI) comprised 28 performance measures assessed by computerised and written tasks and formed a surrogate measure of driving performance (Pass, Borderline or Fail). Baseline characteristics showed 47 patients (74%) had WHO Grade 3 or 4 tumours with majority (98%) managed with surgical resection followed by adjuvant chemoradiation (73%).

Forty-four (69%) participants passed, and twenty (31%) were borderline or did not pass; the latter was combined into one group.In the non-pass group, patients were older (62.6 years vs 49 years, *P* < 0.01), more likely to have tumours of the temporal lobe (60% vs 25%, *P* < 0.02) and higher number of WHO grade 4 tumours (95% vs 63%, *P* = 0.03). Logistic regression analysis showed age and tumour location to be the best predictor of CBDI outcome. The resulting R^2^ value of 0.25, suggests that age and tumour location explained 25% of the total variance of CBDI outcome (*P* = 0.005). Neuropsychological tests were also undertaken to assess cognitive domains important to safe driving. The greatest difference in performance between the pass/non-pass group was within the memory and visual attention domain.

### Comparison of driving guidelines

Driving guidelines of English-speaking countries (Australia, United Kingdom and Canada) were reviewed. No formalised national guidelines regarding driving restrictions in patients with brain tumours exist in the United States.

The included guidelines varied in clarity and depth across different countries (Table 3). Most guidelines had clear recommendations for selected conditions such as seizures and post-intracranial surgery (immediate driving suspension). However, guidelines regarding neurocognitive deficits from intracranial tumours were less clear. The UK guidelines are most comprehensive regarding intracranial tumours, distinguishing benign and malignant tumours, as well as incorporating treatments modalities [7].

**Table 3:** Comparison table of driving guidelines

*Recommendations for private driving*				
Guideline	Space-occupying lesions	Intracranial surgery	Seizures	Vision requirements
Australian^1^	Patients are unfit for unconditional license if there have been significant visuospatial, executive function, cognitive and motor impairments. **Conditional license** may be considered following treating doctor assessment and practical driver assessment.	*Advisory only, non-driving periods may be varied by neurosurgical assessment.* Cease driving for 6 months. If there have been seizures, seizure guidelines also apply	Driving may resume following 6 months following first seizure or newly diagnosed epilepsy treated for the first time. Otherwise, default cessation period of 12 months. 12 month cessation period following a motor vehicle crash.	Visual acuity: Minimum 6/12 on Snellen Chart with one or both eyes. Visual field: - Horizontal extension of visual field must be greater than 110 degrees. - No significant defect in binocular field that extends within 20 degrees of fixation above or below horizontal meridian. - No significant central field loss Conditional license may be considered if condition adequately managed with ongoing reviews and input from treating optometrist and ophthalmologist.
UK^2^	WHO Grade I or II glioma Resume driving 6 months following biopsy if no treatment. If having treatment, resume driving 1 year post completion. WHO Grade III or IV, metastatic deposits, CNS lymphoma Driving may resume 2 years after completion of primary treatment. If treatment is with immunotherapy or targeted therapies, driving may resume one year after completion of therapy provided clinical and imaging evidence of disease stability intra and extracranially. Benign tumours Depends on treatment modality – resume driving 6 months post craniotomy, 1 month post SRS and upon completion of treatment post fractionated radiation therapy. *Infratentorial lesions – driving may resume earlier.*	Benign tumours Resume driving after 6 months provided there is no residual impairment likely to affect safe driving Malignant tumours Driving may resume 2 years after completion of primary treatment.	Driving must cease 6 months from date of seizure of for 12 months if there is underlying causative factor that may increase risk	Visual acuity: - Able to read registration mark fixed to vehicle under current standards and at least Snellen 6/12 with bot or one eye if monocular. Visual field: - Horizontal field of at least 120 degrees without significant defect in binocular field that extends within 20 degrees of fixation above or below horizontal meridian. - Central field loss that only involves scattered single missed points or at most single cluster of up to 3 adjoining points. Exceptions made for consideration of relicensing if defect present for at least 12 months, non-progressive pathology without other impairment of visual function.
Canadian^3^	Malignant No general recommendation can be made following resection of malignant brain tumour. Assessment from consulting neurologist and surgeon should be sought. Benign No driving restrictions if there are no cognitive, visuospatial, motor or executive function impairments post primary treatment. However, defer to seizure guideline if seizure has occurred before or after resection of the tumour.		Cease driving for at least 3 months and require neurological assessment. If seizure has occurred before or after tumour removal, need 12 months seizure-free period before resuming driving.	Visual acuity At least Snellen 6/15 with both eyes examined together. Visual field 120 degree continuous along horizontal meridian and 15 degrees continuous above and below fixation. Exceptions can be made in cases where deficits have been longstanding but will require special visual assessments.

^1^AustRoads – 2022

^2^Canadian Medical Association Driving Guidelines – 9th edition 2019

^3^United Kingdom Driving Guidelines – May 2022

Despite distinguishing benign and malignant tumours, Canadian guidelines advised against generalised recommendations for malignant tumours, instead advocating for assessments by the patients’ treating neurologist or neurosurgeon [6]. Australian guidelines were predominantly situationally-focused with recommendations including immediate driving cessation post-intracranial surgery, with duration determined by the neurosurgeon and 6 to 12 months’ cessation for seizures depending upon risk factors [8]. All guidelines address visual deficits requiring acuity and field clearance but did not consistently address other neurological deficits.

## Discussion

To our knowledge, this is the first systematic review evaluating driving in patients with brain tumours and incidence of MVC. To date, most published research has focused on clinician attitudes and practices towards driving guidelines [15–19]. Our review demonstrates a paucity of real-world data concerning MVC incidence amongst these patients. Of the three studies identified, only one study directly evaluated MVC incidence, rather than a surrogate marker. Whilst no significantly increased risk of MVC was reported by these studies Mansur et al. revealed that almost 40% of patients claimed to have had a MVC following diagnosis, suggesting discordance between real-world data and the driving risk assessment tools used [13]. Data interpretation is limited by small sample size, lack of a control population, heterogenous tumour types and selection bias surrounding coexistence with seizures. Nevertheless, all studies underline the neurological complexities involved in safe driving and suggest the need for more comprehensive and specific cognitive assessments and guidelines for patients driving with brain tumours.

The responsibility of determining a patient’s medical fitness to drive often falls on the treating clinician. However, surveys globally have consistently highlighted that most clinicians are unaware of driving guidelines [15–21]. In an Australian survey of 194 practicing neurosurgeons, radiation oncologists and neurologists, 73% of respondents were not aware of guidelines and only 38% of doctors discussed driving restrictions with patients [20]. North American studies have reported similar findings [15, 17, 18].

Additionally, providers familiar with driving guidelines, report significant heterogeneity in their application [20]. A Canadian study evaluating driving recommendations among healthcare professionals, found that only 56% discussed driving recommendations with patients and only 9.3% could reliably determine fitness to drive [18]**.** In a survey of Australian and New Zealand palliative care clinicians, only 27% reported patients with brain tumours to driving authorities [19]**.** Similarly, a Canadian study demonstrated low mandatory reporting rates with poor documentation of medical discussions regarding safe driving [15]. There are likely several factors that contribute to this diverse practice and reluctance to report, including lack of awareness of guidelines, limited tools and training to determine fitness-to-drive, and intent to preserve patients’ autonomy [22]. Altogether, these findings re-affirm the need to develop more comprehensive and specific driving recommendations at a global level.

Furthermore, driving recommendations from governing authorities do not address the clinical issues patients with brain tumours typically face. Specific guidelines addressing seizures and time since surgery exist, but there is little emphasis on impaired neurocognitive and motor functions from disease progression and treatment toxicities. A pilot study of neurocognitive function following radiation therapy to one to three brain metastases revealed baseline deficits in learning/memory, motor dexterity and higher executive function [23]. Additionally, one month after stereotactic radiosurgery, 54% of patients declined in two or more domains; especially motor dexterity and learning/memory. Thus, whilst current guidelines offer clear recommendations for specific situations such as seizures, they inadequately address neurocognitive deficits related to disease progression or treatment toxicities.

Aside from UK guidelines, Australian and Canadian guidelines do not consider actual tumour characteristics. Though the studies by Mansur et al. and Estevis et al. did not find correlation between tumour histology or location with driving performance; studies have shown some difference in symptomatology and risk of progression that may impact on MVC risk. Lower grade gliomas have higher frequency of seizures; conversely malignant tumours have lower incidences due to its rapid and destructive growth [2, 3]. Tumour location can impact on syndrome of neurological deficits, such as posterior fossa tumours which are associated with cerebellar signs, cranial nerve palsies and raised intracranial pressures [24]. As such, these factors need to be reflected in driving guidelines.

Driving is complex, involving several neurocognitive domains and therefore requires a multidisciplinary approach to support clinicians in balancing competing patient advocacy and community safety responsibilities. As demonstrated in Mansur’s study, no single cognitive test can predict driving performance [13]. A meta-analysis of driving assessments of patients with Alzheimer’s dementia and mild cognitive impairment show that measures of cognitive and sensory domains may be predictive of driving performance with the TMT test (assess attention, processing speed and adaptability) and the Maze Task (measures executive planning and visuospatial awareness) being better predictors of on-road performance than driving simulations [25]. Mansur’s study included cognitive assessments with both tests, however they were not predictive of MVC. Therefore, cognitive testing alone in assessments of ‘fitness-to-drive’ is inadequate. This further highlights the need for a more comprehensive guide and assessment for clinicians on evaluating patient’s fitness-to-drive.

To address the gap in driving assessment tools, the Swiss Neuro-Oncology Society in collaboration with the Swiss Society for Legal Medicine have developed a guideline involving routine brain MRI, thorough history, neuropsychological and visual assessments to determine ‘fitness-to-drive’ in patients with glioblastoma following their initial treatment [26]. This group is now conducting a pilot project prospectively recruiting patients with glioblastoma who wish to return to driving, to determine feasibility of this proposed tool in daily clinical practice and to match outcomes with events from the Road Traffic Registry (GLIODRIVE). Beyond this and perhaps more importantly, is the development of driver rehabilitation programs to re-introduce patients to driving.

Several limitations should be considered of this review. Few studies of fair quality were included. These studies were heterogeneous in design, methodology and outcomes, making comparisons challenging and limiting conclusions drawn. Additionally sample sizes across the three studies were small. Another major limitation of all three studies is that they do not consider the dynamic course of brain tumours, and the risk of disease progression and thus, development of new deficits or complications. This is important to consider when developing guidelines and assessment frameworks. Whilst the gold standard test for assessing driving ability is an on-road driving assessment, only one study looked at real-world driving outcomes.

Future research should include registry studies that examine real-world data of impact of brain tumours on MVC risk and explore what factors may increase MVC risk such as disease characteristics (tumour histopathology, location), treatment modalities and patient characteristics. Studies examining neurocognitive domains important to safe driving and how they are affected in brain tumours is also important to guide development of driving assessment tools. Addressing these gaps in literature is required to form evidence-based driving recommendations.

## Conclusion

Overall, there is limited data regarding MVC risk and driving behaviours in patients driving with brain tumours.

Though there was no significant difference in MVC risk observed in the studies included, our review has highlighted neurocognitive deficits in domains important to safe driving in this group. Current driving guidelines inadequately address these deficits and do not reflect the dynamic nature of disease with progression and treatment toxicities. Further work is required to explore driving behaviours and risk in patients with brain tumours to guide formation of driving assessment tools and frameworks to allow for development of more comprehensive guidelines that balance patient autonomy and community safety.

### Supplementary Information

Below is the link to the electronic supplementary material.

Supplementary file1 (DOCX 23 KB)

## Data Availability

No datasets were generated or analysed during the current study.
