# Supplementary material for: A systematic review of the impact of brain tumours on risk of motor vehicle crashes
Source: J Neurooncol. 2024 Feb 6;166(3):395–405. doi: 10.1007/s11060-024-04586-6 (PMC10876497; doi:10.1007/s11060-024-04586-6)
Supplement: Supplementary file 1 — Supplementary file1 (DOCX 23 KB) [file 11060_2024_4586_MOESM1_ESM.docx]

**Supplementary Information**

**Supplementary File 1:** **Criteria for the Newcastle-Ottawa Scale regarding star allocation to assess quality of studies (out of a total of nine stars)**

| **Criteria** | **Acceptable (star awarded):** | **Unacceptable (star not awarded):** |
| --- | --- | --- |
| *Representativeness of exposed cohort* | Population-based | Hospital-based |
| *Selection of non-exposed cohort* | Same setting as exposed cohort | Different setting from exposed cohort |
| *Ascertainment of exposure* | Secure records or directly measured | Self-reported information |
| *Comparability* | Excluded or adjusted for prior outcome in analysis  Adjusted for age, gender, tumour type, tumour location | No exclusion of  Did not adjust for age, gender, tumour type, tumour location |
| *Outcome of interest* | Secure records or directly measured | Self-reported information |
| *Adequacy of follow-up*  Was follow up long enough for outcomes to occur?  Complete follow up - ?all subjects accounted for | Adjusted for missing data or follow-up | No statement regarding missing data |

**Supplementary File 2: OvidMEDLINE Search Strategy**

*1974 to 7 March 2023*

|  | **Search** | **Results** |
| --- | --- | --- |
| 1 | Brain neoplasms/ or cerebral ventricle neoplasms/ or infratentorial neoplasms/ or brain stem neoplasms/ or cerebellar neoplasms/ or neurocytoma/ or supratentorial neoplasms/ or hypothalamic neoplasms/ or pituitary neoplasms | 250502 |
| 2 | Glioma/or astrocytoma/ or glioblastoma/ or diffuse intrinsic pontine glioma/ or ependymoma/ or glioma, subependymal/ or ganglioglioma/ or gliosarcoma/ or medulloblastoma/ or oligodendroglioma/ or optic nerve glioma/ or neurocytoma | 279371 |
| 3 | ((brain* or cerebral or cerebel* or intracranial or intra-cranial) adj3 (cancer* or malignan* or neoplasm* or metastas* or tumo?r*)).mp. | 379415 |
| 4 | ((infratentorial or supratentorial or hypothalamic or pituitary) adj (cancer* or malignan* or neoplasm* or metastas* or tumo?r*)).mp. | 52243 |
| 5 | (glioma* or astrocytoma* or glioblastoma* or ependymoma* or ganglioglioma* or gliosarcoma* or medulloblastoma* or oligodendroglioma* or neurocytoma*).mp. | 350828 |
| 6 | 1 or 2 or 3 or 4 or 5 | 624059 |
| 7 | Accidents, Traffic/ | 110087 |
| 8 | ((Traffic or road* or freeway* or highway* or car or cars or vehicle* or vehicular or automobile* or bus or buses or truck* or bicycle* or motorbike* or motorcycle* or motor bike* or motor cycle*) adj (Accident? Or crash* or collision*)).mp. | 118445 |
| 9 | automobile driving/or automobile driver examination/ or distracted driving/ | 40439 |
| 10 | ((drive or driver* or driving) adj3 (fit or fitness or safe* or restrict* or behavio* or habit? Or simulation* or simulator* or perform* or assess* or recommend* or regulation* or skill* or school* or train* or education* or test* or licen* or impair* or advice or advising or exam* or eligibility* or eligible or ability* or inability* or ineligible or unfit or difficult* or dangerous*)).mp. | 64330 |
| 11 | ((Traffic or road? Or freeway* or highway* or car* or vehicle* or vehicular or automobile* or bus* or truck* or bicycle* or motorbike* or motorcycle* or motor bike* or motor cycle*) adj2 (driving or driver* or riding or rider*)).mp. | 54189 |
| 12 | 7 or 8 or 9 or 10 or 11 | 215979 |
| 13 | 6 and 12 | 800 |
| 14 | 13 not (gene* or epigene* or cancer drive*).mp. | 534 |
